# Response of Ecosystem Services to Land Use Change in Madagascar Island, Africa: A Multi-Scale Perspective

**DOI:** 10.3390/ijerph20043060

**Published:** 2023-02-09

**Authors:** Flaubert Tiandraza, Shijin Qu, Shougeng Hu, Christopher N. Mkono, Anna Tikhomirova, Solo Nirina Randrialahamady

**Affiliations:** 1 Department of Land Resource Management, School of Public Administration, China University of Geosciences, Wuhan 430074, China; 2 Key Laboratory of Rule of Law Research, Ministry of Natural Resources, Wuhan 430074, China; 3 Key Laboratory of Theory and Technology of Petroleum Exploration and Development in Hubei Province and Key Laboratory of Petroleum Resources, China University of Geosciences, Wuhan 430074, China; 4 School of Economics and Management, China University of Geosciences, Wuhan 430074, China; 5 Department of Law, Economics, and Management at the Management Mention Finance and Accounting Course, University of Toamasina, Toamasina 501, Madagascar

**Keywords:** ecosystem service value, benefits transfer, sensitive analysis, Madagascar island, agricultural expansion, land use and land cover change (LULCC)

## Abstract

“Land Use and Land Cover Change (LULCC)” is increasingly being affected by ecosystem services value. LULCC patterns have been subjected to significant changes over time, primarily due to an ever-increasing population. It is rare to attempt to analyze the influence of such changes on a large variety of ecosystem benefits in Madagascar island. The economic value of ecosystem services in Madagascar island is evaluated throughout the period from 2000 to 2019. The expansion of the human population affects the changing value of ecosystem services directly. The PROBA-V SR time series 300 m spatial resolution cover of land datasets from the “Climate Change Initiative of the European Space Agency (ESA)” were used to measure the values of ecosystem activities and the changes in those values caused by land use. A value transfer method was used to evaluate the value of ecosystem services to land use changes on Madagascar island. The findings show that from 2000 to 2019, at the annual rate of 2.17 percent, Madagascar island’s ecosystem service value (ESV) grew to 6.99 billion US dollars. The components that greatly contributed to the total change of ESV were waste treatment, genetic resources, food production, and habitat/refugia. These components in 2000 contributed 21.27%, 20.20%, 17.38%, and 13.80% of the total ESV, and 22.55%, 19.76%, 17.29%, and 13.78% of the total ESV in 2019, respectively. Furthermore, it was found that there was a great change in LULCC. From 2000 to 2019, bare land, built-up land, cultivated land, savannah, and wetland increased while other LULCC types decreased. The sensitivity coefficient ranged from 0.649 to 1.000, <1, with forestland registering the highest values. Wetland is in the second position for the most important land cover category in Madagascar, considering the total value of the ecosystem. The value of ecosystem benefits per unit of the land area was higher on cultivated land, despite the relatively low fraction of cultivated land area across these eras. The sensitivity indices of seven land types from 2000 to 2019 were mapped to understand better the geographical distribution patterns of ESV’s “equivalent value coefficient” (VC) across various land uses. It is suggested that the ESV should be included in Madagascar’s government land-use plan to manage it effectively and efficiently with fewer negative effects on the ecosystem.

## 1. Introduction

Ecosystem services value (waste assimilation) and goods (food) fall under the category of ecosystem services, which refers to the benefits gained from ecosystem functions [1]. Nevertheless, services, including cultural services (such as aesthetic and recreational values), regulatory services (such as water purification and climate regulation), and provisioning services (such as fuel, food, and water) have a direct effect on people’s lives while supporting services (such as erosion control and soil foundation) have a more indirect effect [2,3,4]. Additionally, land use and land cover changes have affected these services. While humans have been modifying the earth for thousands of years to produce food, fiber, fuel, and other necessities, the current rate, scope, and severity of change are unprecedented compared to the past [5]. Due to rapidly expanding populations, development of the economy, and urbanization, LULC has changed dramatically in recent decades, shifting from the woods, savannahs, and other native lands to croplands, pastures, and urban regions [6,7]. By 2050, the increase in agriculture areas, mostly from forests, is expected to reach 13 million ha/yr [2]. Furthermore, around 40% of the planet’s surface is cultivated or used for grazing [8]. Due to the influence of LULC on natural ecosystems, they have become less capable of supplying goods and services to society in the present and the future [4,9]. Planting trees, cultivating crops, and building urban areas have led to enormous increases in food production, fiber, and timber, as well as housing and other things worldwide; report shows that the increase in biodiversity has caused 60% of ecosystem services to decline during the last five decades [10]. Global concerns are rising regarding minimizing the negative impacts of urbanization and economic development on the world’s natural ecosystems and the products and services that ecosystems produce [11]. Despite this, most ecosystem services they provide are regarded as public goods, which the market does not capture. The economic value of ecosystems is often not fully recognized, which can lead to unsustainable land use decisions. The objective comparison of the potential impacts of different land use options and decision-making that promote sustainable land use can be performed by the assessment of the ecosystem services value [12,13]. By understanding and valuing the benefits that ecosystems provide, more informed choices that balance economic development with the protection of biodiversity and the provision of essential ecosystem services can be made [4,14].

Understanding the way ecosystem services are provided and valued at a multi-scale level is necessary to link environmental protection with human prosperity. Indeed, population dynamics are an important factor having strong environmental impacts [11,15,16,17]. Most experts concur that a solid understanding of the direct and indirect ways various land-use and land-cover (LULC) components’ give goods and services to people is essential to efficiently manage and protect the services provided by nature [6,18,19]. Therefore, the knowledge of the processes involved in classifying LULC over place and time must be improved [20]. Standard approaches to value ecosystem services highlight this fact by estimating the value of specific services in a given area and then extrapolating those values to similar regions with similar habitat types [21]. Determining geographic patterns of ecosystem service value (ESV) is more complicated due to the number of classification algorithms used to create LULC datasets [22]. There have also been numerous attempts to put a monetary value on ecological services. Although it is plausible to know which LULC categorization or economic valuation procedures may be more accurate, some may be more suitable under certain circumstances [20]. Accessibility might vary depending on factors such as available time, money, and level of expertise. As a result, the best methods may not always be used, which can lead to inaccuracies or inappropriate choices being made. Therefore, it is crucial to comprehend how alternative valuation strategies influence the outcomes upon which management decisions are based [23].

Various methods have been discussed in the literature, such as market prices and trip costs, expressed preferences (such as choice experiments and contingent values), approaches based on cost (such as substitution and preventable costs), plus benefit transfers [24]. A secondary valuation method, the Benefits Transfer Method (BTM), acclimates formerly established estimates based on original assessment studies in various locations to regions with similar ecological, economic, and demographic characteristics [25].

Since the 1990s, BTM has been broadly utilized in numerous environmental policy and natural resource contexts, including water quality management. Since the 1990s, health risk assessments for water quality, waste management, and forest management have been conducted [26,27,28]. BTM calculated the worldwide economic worth of 16 biomes’ 17 ecosystem services. An expanded database of over 300 case studies worldwide was used to obtain the updated estimates. Changes in ecosystem services value could be analyzed using underlying data and algorithms at different scales [1,12].

Even though human actions affect ecosystems all over the world [29], the effects are most noticeable in the tropics [14], where agriculture is vital [30,31]. Tropical rainforests have the most biological diversity on land; however, they are continually being destroyed.

Only a few studies on LULC change have been conducted across Madagascar’s provinces [32], indicating a lack of national information on LULC change. Furthermore, a quantitative assessment showing the ecosystem service value changes is rarely assessed, mostly covering the whole country, including provinces, regions, and towns [33]. In addition to examining the changing characteristics of LULC in Madagascar, there are no local or national estimates of these consequences which is essential information required in this kind of study [34]. Therefore, this study represents the first data about linking land use change to loss/gain in ecosystem services in Madagascar island.

It is well-known that the monetary worth of ecosystem services changes gradually with time. Therefore, in this investigation, the change in spatial variations over time has been taken into consideration. The present study employs methods to evaluate various management zones of Madagascar island based on the ecosystem services they provide. Key management areas were pinpointed and ranked in order of priority. Management plans to protect the unique biodiversity of Madagascar island and consider the needs of the people who live in the provinces where the biodiversity is found. It, therefore, provides a highly relevant case study for assessing the impact of the implementation of different methods to measure nature’s utility to society. In addition, this study intends to fill up the knowledge gaps left by prior studies on LULC change by using data from Land-Use and Land-Cover Changes in Madagascar island from 2000 to 2019. Furthermore, the present research attempted to answer two primary questions: (1) What happened to LULC in Madagascar island between 2000 and 2019? (2) How did this period’s LULC dynamics affect the values of the ESV and the other ecosystem functions?

This study hypothesizes that changes in LULC patterns significantly affected the deterioration of natural ecosystem service functions over time. Overall, ESV across administration zones was compared using the current value over a given time frame and across space and time on a multi-scale level. By using these methods, this paper could show ecosystem managers the results that can be obtained from the available data. In light of this astronomical expansion in the human population, the already-stretched limit of finite land resources will put additional strain on the country’s environment and raw forest resources.

## 2. Materials and Methods

### 2.1. Study Area

**Figure 1:**
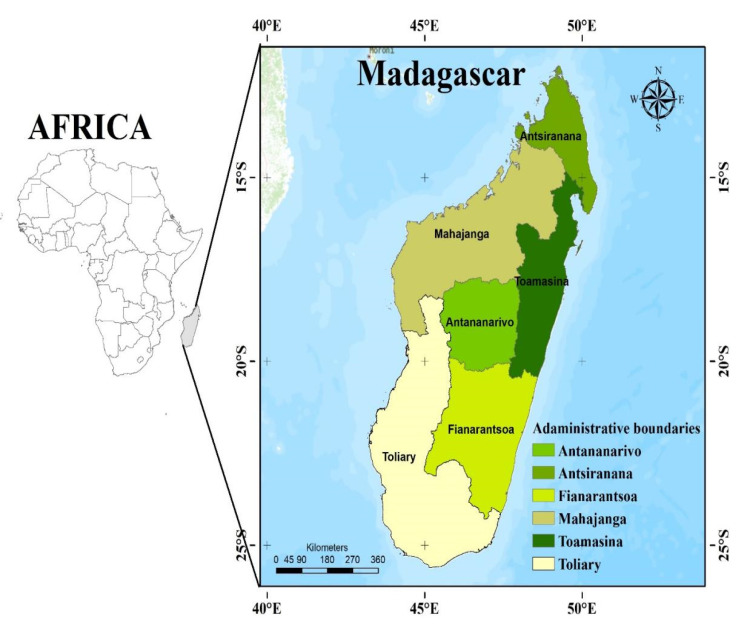
Map showing the location of major provinces in Madagascar and geographical zones (Source: [38,39]).

Madagascar, the world’s second-largest island nation, is one of the megadiverse countries with a high concentration of endemic species, located in the Indian Ocean between latitudes 22° 66′ and 23° 31′ S and longitudes 43° 46′ and 44° 05′ E (Figure 1). The island is bordered on the west by the Mozambique channel and the east by the Indian Ocean, with a total size of around 592,800 square kilometers [35]. The island’s ecosystems include reefs, drylands, mangroves, wetlands, lakes, rivers, steppes, savannahs, and forests [35].

Madagascar’s population increased from 15.77 million to 26.97 million between 2000 and 2019. In 19 years, the population expanded by 11.2 million (71%), demonstrating significant population expansion [36]. The ecological environment is deteriorating, and environmental resources are diminishing as population and land urbanization continue to expand.

Madagascar island has two distinct seasons: the hot and rainy season, which goes from November to April, and the colder and dryer season, which extends from May to October. Along with receiving the highest and most regular rainfall, the east coast receives a maximum of 3700 mm of rainfall each year. The climate on the east coast is sub-equatorial, and easterly trade winds influence it. The western coast of the States is often drier than the rest of the country and suffers from a substantial level of coastal erosion. The southwest and the extreme south are classified as semi-arid regions since they receive less than 800 mm of precipitation yearly. On average, temperatures along the coast range from 23 °C to 27 °C, while temperatures in the interior mountains range from 16 °C to 19 °C [37].

### 2.2. Data Sources

The European Space Agency-Climate Change Initiative (ESA CCI) provided data about land cover between 2000 and 2019. Information on long-term variations in land cover from 2000 to 2019 is shown in the ESA CCI’s land cover products at a 300 m resolution and is based on yearly data [40]. Thirty-seven original land cover types described in this dataset (UN-LCCS) are classified by applying the “United Nations Land Cover Classification System” [40,41]. Recent years have seen a rise in the number of case studies that use this dataset to investigate the accessibility of information regarding land cover [42,43]. In total, 2600 primary sample units and an object-based validation database were created to assess land cover accuracy [40], with a weighted-area accuracy of 71.1% [44]. Specifically, among the coarse-resolution datasets, the highest overall accuracy was Madagascar’s ESA CCI land-cover data [45,46]. Thus, ESA CCI land cover data can offer insightful information for specified locations in Madagascar. The nineteen-year land cover maps are analyzed in this study using 19 different categories. Table 1 shows Madagascar’s land cover classification.

Madagascar, with the 16 biomes identified by [12] and the closest matching biome, was utilized as a surrogate (Table 1). Cultivated land was utilized by cropland; forest by tropical forests; scrublands and grassland for rangelands and grass; built-up land for urban areas; and the bare regions for desert, tundra, ice, and rock. Savannahs are the collective term for grasslands and scrublands, which occupy the same comparable biome (Table 2).

### 2.3. Methods

**Figure 2:**
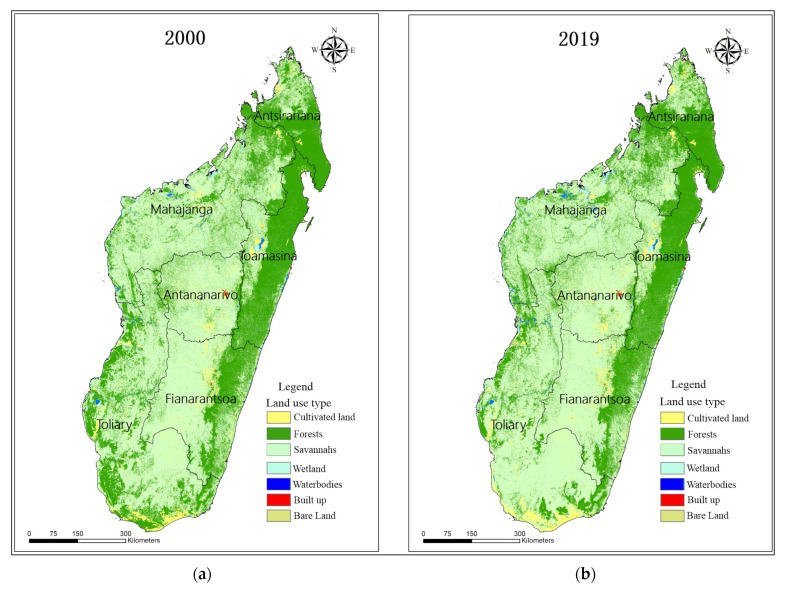
Madagascar island LULC spatial distribution (**a**) 2000 and (**b**) 2019.

The methodology is represented in Figure 2, and the sensitivity of ESV to LULCC was analyzed.

#### 2.3.1. Ecosystem Service Assigned Values

The BTM-based value coefficients employed were used by Costanza et al., 2014 [12]. Their valuation coefficients were utilized for three main reasons in our study. First, they estimated 17 ecosystem service functions and 16 major biomes, making them the most comprehensive value coefficients. Second, valuation studies in Madagascar mainly concentrated on the inland wetland biome [47]. There is an economic estimate available for providing services. However, the estimation is likely to understate the biome’s economic value because wetlands supply a significant number of additional services that are highly valued [12] as well as recognized in Madagascar [48,49]. Finally, due to the limitations imposed by the scope of our investigation, we could not make any estimates for the ESV total for additional biomes. We contrasted the eight forms of LULC found in Madagascar (Table 1) with the 19 different biomes reported by [12], and the biome that is closest in comparison was used as a surrogate.

#### 2.3.2. Calculation of ESV

Constanza’s ESV evaluation model is used as well as other studies to compute the overall ESV in Madagascar and each state [50,51]:

(1) $$ESV_{t}=\sum_{k=1}^{n}\left(A_{kt}\times VC_{k}\right)$$

where $VC_{k}$ is the ecosystem services value coefficient (US$ha^−1^ yr^−1^) of the LULC Type k, $A_{kt}$ is the area (ha) of the LULC type $A_{kt}$ at a time, and $ESV_{t}$ is the estimated total ESV at the time *t*.

ESV time change was computed with the formula;

(2) $$ESV_{cr}=\frac{\left(ESV_{t_{2}}-ESV\right)}{ESV_{t_{1}}}\times 100\%$$

During the observation period $t_{1}$ to $t_{2}$, ESV’s change rate is $ESV_{cr}$, at the conclusion and start of the observation $t_{2}$ and $t_{1}$ the total estimated ESV is $ESV_{t_{2}}$ and $ESV_{t_{1}}$, respectively.

To determine LULC alterations effect on every ecosystem function, the value of each ecosystem function in terms of the services it provides is computed with the formula:

(3) $$ESV_{ft}=\sum_{k=1}^{n}\left(A_{kt}\times VC_{fk}\right)$$

where $ESV_{ft}$ is the estimated ESV of function f at time *t*, $A_{k}$ is the area (ha) for the LULC type k, and $VC_{k}$ is the coefficient of ecosystem service value of the function (US$ha^−1^ yr^−1^) for the LULC type k.

#### 2.3.3. Sensitivity Analysis

As an essential component of the ESV estimating process, we used a sensitivity analysis to effectively validate the elasticity between the “total ESV” and the “equivalent value coefficient (VC)” of different types of land use [52,53]. If both the VC’s elasticity and the evaluation outcome are reliable, an elasticity number greater than one would indicate that ESV was elastic with respect to VC.

The idea of elasticity from the discipline of economics was utilized in this research to determine the degree to which the overall ESV is sensitive to the local VC of various land uses [54]. Adjustments of up to fifty were made to the corresponding parts of each of the seven land use types to determine how much ESV depends on VC factors. The particular equation for the computation is represented below:

(4) $$CS=\left|\frac{\left(ESV_{j}-ESV_{i}\right)/ESVi}{\left(VC_{jk}-VC_{ik}\right)/VC_{ik}}\right|$$

CS denotes the sensitivity coefficient in this Equation (4), *ESV_i_* and *ESV_j_* denote the value of the ecosystem service before and after adjustment, respectively, while the *VC_ik_* and *VC_jk_* denote the equivalent value factor of the kth land use type consecutively before and after adjustment.

## 3. Outcomes

### 3.1. Land-Use/Land-Cover(LULC) Pattern in Madagascar Island 

Figure 2 shows the spatial distribution of Madagascar’s LULC patterns in 2000 and 2019. In the LULC categorization, Savannahs occupied the most territory in 2000, making up over 58.20% of the total area [41]. Agriculture and forests also have large areas, making up 13.4% and 25.8% of Madagascar’s total land area. The other LULC types (bare land, built-up land, water bodies, and wetlands) only made up 2.6% of Madagascar’s total land area (Table 3).

### 3.2. Land-Use/Land Cover(LULC) Change in Madagascar Island from 2000 to 2019 

Bare land, built-up land (savannah, agriculture), and wetlands increased from 2000 to 2019, whereas other classes of LULC decreased (Table 3). At an annual rate of 0.21%, 317.94 ha of cultivable land was developed during the research period, 25.8% less than the bare land in 2000. At an annual rate of 0.02%, the savannah expanded to approximately 158.66 ha, the wetland to approximately 55.69 ha, the built-up area to approximately 14.67 ha, and the undeveloped land to about 9.14 ha. It is estimated that aquatic bodies lost 11.4% of their initial surface area in 2019, while woods reported losses of 3.26%.

### 3.3. Estimated Changes in Ecosystem Services on Madagascar Island

#### 3.3.1. Change in total ESV from 2000 to 2019 

According to [12], the value of Madagascar’s ecosystem services (ESV) was assessed at 364.88 billion dollars in US dollars in 2000 (Table 4, Table 5 and Table 6). Savannah was the most significant contributor to this total (about 34.49%), and it was followed by wetlands, forests, and cultivated land, which contributed 24.01%, 22.63%, and 12.14%, respectively. Water bodies and white built-up lands were 1.67% and 0.006% of the total ESV, respectively.

From 2000 to 2019, ESV increased the natural ecosystem in Madagascar island by 5.41%, from US $320.37 billion to US $325.49 billion per year (Table 4, Table 5 and Table 6). This natural landscape’s aggregate ESV gain, charged in the wetland for the total ESV loss, decreased by 13.78% and 152.50%, followed by Savannah with 13.86%. In comparison, water bodies and forests contributed to the total ESV loss with a decrease of 13.78% and 52.6%, respectively. A comparison of artificial ecosystems (i.e., built-up and cultivated land) shows that the overall ESV increased by 4.37%, from US $2.34 billion to US $2.44 billion per year, with 97.38% of that growth coming from cultivated land. From 2000 to 2019, Madagascar’s ESV grew by US $6.99 billion, or 2.17% yearly.

The administrative provinces show that ESV was highest for Mahajanga province (92.12 billion US $), Fianarantsoa province (US $51.82 billion), Toamasina province (US $47.17 billion), Antsiranana province (US $32.42 billion) and Antananarivo province (US $26.59 billion). Savannah largely contributed the ESV for these provinces in Mahajanga (37.89%), Toliary (48.70%), Fianarantsoa (47.02%), and Antananarivo (79.41%); Forests in Toamasina (38.51) and Antsiranana (32.74%); Cultivate land in Toamasina (28.51%) Antananarivo province was the one with lowest ESV (US $26.59 billion) mainly caused by Savannah (21.11%). The rate of change in ESV increased from 2000 to 2019 in all six provinces. Mahajanga province shows the highest ESV rate (2.91%). Followed by Toliary (2.01%), Toamasina (1.97%), Antsiranana (0.71%), Fianarantsoa (0.50%) and Antananarivo (0.20%).

**Figure 3:**
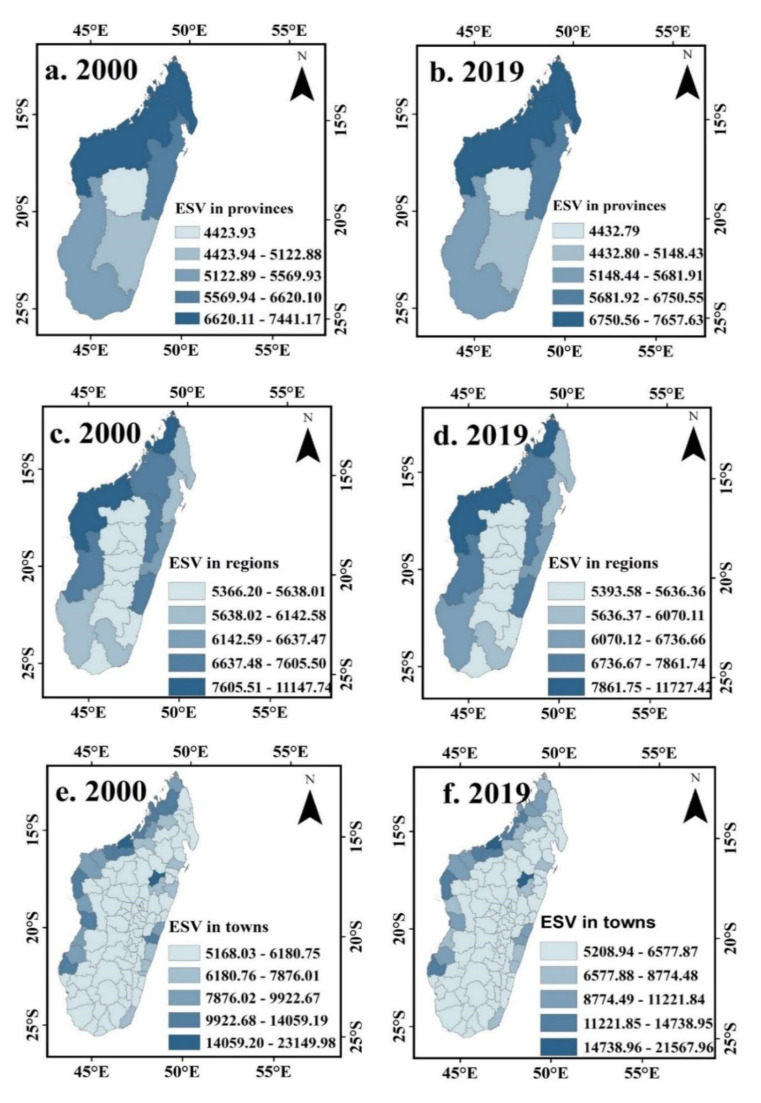
Spatial pattern of change in the rate of ecosystem services value for the provinces (**a**,**b**), the regions (**c**,**d**), and towns (**e**,**f**) in Madagascar island from 2000 to 2019.

**Figure 4:**
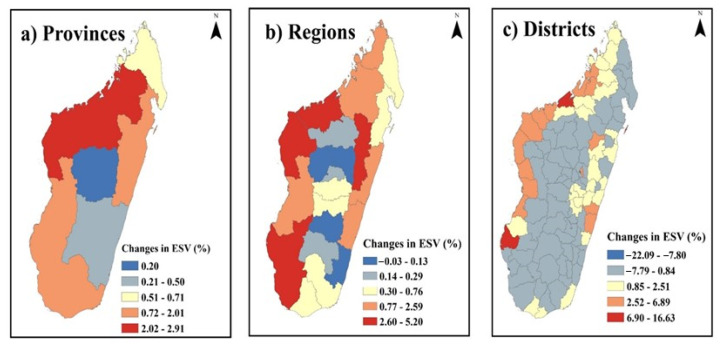
Spatial pattern of change in the rate of ecosystem services value (%) in different scales for the provinces (**a**), the regions (**b**), and towns (**c**) in Madagascar island from 2000 to 2019.

Figure 3c,d shows the spatial pattern of ESV per unit area at the regional scale in Madagascar Island for the period from 2000 to 2019. Generally, the high-value area of ESV has mainly located in the northern part of the study region as well as some parts on the east side of the region. The central and southern parts of the study area have low ESV. The same result can also be observed in Figure 3e,f, which show the spatial pattern of ESV per unit area at the town scale in Madagascar island for 2000–2019, where Mahajanga town, in the north-west part and Amparafaravola town, in the east part of the island, has the highest ESV value among all towns, while Mahabo town, located in the nearly central part of the island, has the lowest ESV value. Figure 4 shows Spatial pattern of change in the rate of ecosystem services value (%) in different scales for the provinces, regions and towns in Madagascar island from 2000 to 2019.

#### 3.3.2. Changes in Values of Ecosystem Functions in Madagascar Island from 2000 to 2019

The study elaborates on and compares the contribution of every ecosystem function to the total ESV in Madagascar island (Table 7). Results show that the most important components contributing to the total ESV in Madagascar island between 2000 and 2019 were waste treatment, genetic resources, food production, and habitat/refugia. These components in 2000 contributed 21.27%, 20.20%, 17.38%, and 13.80% of the total, and 22.55%, 19.76%, 17.29%, and 13.78% of the total in 2019, respectively. From 2000 to 2019, there was an overall change that resulted in an increase in all ecosystem service functions, excluding water regulation, pollution, regulation of climate, regulation of gas, genetic resources, and leisure, which decreased by −0.92%, −2.38%, −6.42%, −0.3%, and −1.82%, respectively. The total ESV of waste treatment increased very sharply than other ecosystem services (8.02%), followed by nutrient cycling (7.53%), disturbance regulation (5.74%), soil promotion (3.60%), habitat/refugia (1.72%), the raw material (1.14%) and the water supply (1.06%). The rate of Charge $ESV_{f}$ increased very slowly for cultural (0.98%).

#### 3.3.3. Sensitivity Analysis of Ecosystem Services Value in Madagascar island from 2000 to 2019 

Table 8 illustrates the sensitivity of ecosystem service values for Madagascar. The sensitivity coefficient ranged from 0.649 to 1.000, always remaining below 1 (i.e., forestland). Because the worth of ecosystem services is independent of the sensitivity coefficient, the estimation results can be considered credible. The sensitivity coefficient was highest (0.649) for forestland in 2000 to the significant amount of forestland and a high ecosystem service value coefficient per unit area, which means that a 1% increase in Madagascar’s forestland area would result in a 0.649% growth in the projected worth of ecosystem services. Forest land generally had a high variation of sensitivity coefficient than other land uses. The island’s forest cover mainly drives the value of Madagascar’s ecosystem services. Wetland is Madagascar’s second most noteworthy cover category considering the entire environment esteem. Cultivated land provided more ecosystem benefits per unit of land area than uncultivated land, despite the relatively low percentage of cultivated land across these periods.

**Figure 5:**
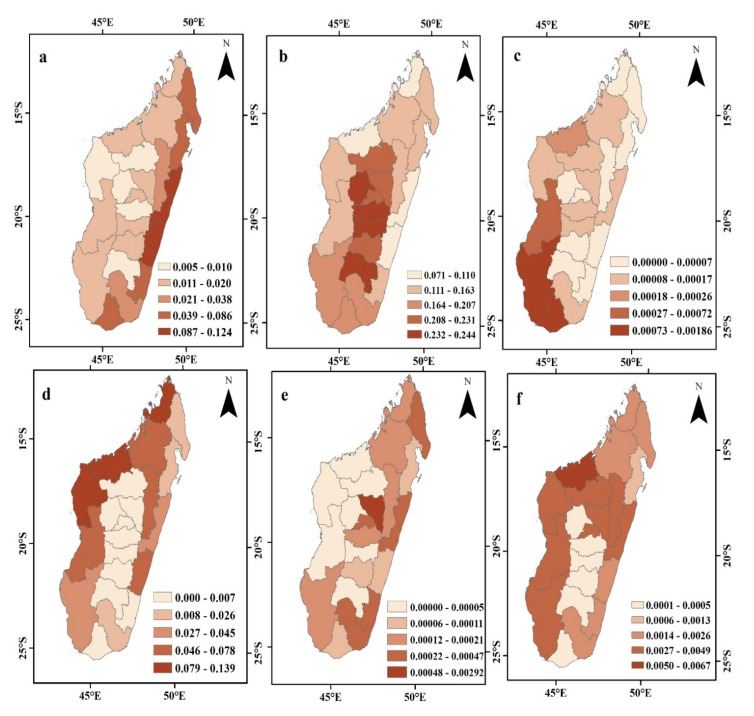
Spatial pattern of sensitivity coefficients of different land-use types in Madagascar island in 2019. Important factors were (**a**) the spatial pattern of the sensitivity coefficient of cultivated land; (**b**) the spatial pattern of the sensitivity coefficient of forestland; (**c**) the spatial pattern of the sensitivity coefficient of the savannah; (**d**) the spatial pattern of the sensitivity coefficient of wetland; (**e**) the spatial pattern of the sensitivity coefficient of built up; (**f**) the spatial pattern of the sensitivity coefficient of water.

The sensitivity indices of seven land types from 2000 to 2019 were mapped to understand better the geographical distribution patterns of ESV’s “equivalent value coefficient” (VC) across various land uses. Although, ESV’s sensitivity index to VC of various land-use categories followed a consistent spatial distribution pattern over the years (Figure 5). It was discovered that farmed land in the plains poses a substantially greater sensitivity index than land in the mountains (Figure 5a).

This is because the ESV was more susceptible to fluctuation in cultivated land, which was abundant in the plains. Forest and savannah sensitivity indices in the surrounding mountainous areas were relatively higher in the Bongolava region, Vakinakaratra region, Amoron’i mania region, Ihorombe, and Atsimo Andrefana regions, Androy region (Figure 5b,c). Both the wetland and water area sensitivity indices in and around the Diana, Melaky, and Boeny regions were much higher than in other regions (Figure 5d,f). The Analamanga region, urban agglomerations, and the suburbs and exurbs of major cities were the most common locations where built-up land sensitivity indexes were high (Figure 5e). For regional ecological protection measures and land-use planning, as well as for providing more scientific guidance for optimizing the land use structure and the coordinated improvement of ecology and economy on the Madagascar island, analyzing the sensitivity indices of various land use types in various county units was of great significance.

**Figure 6:**
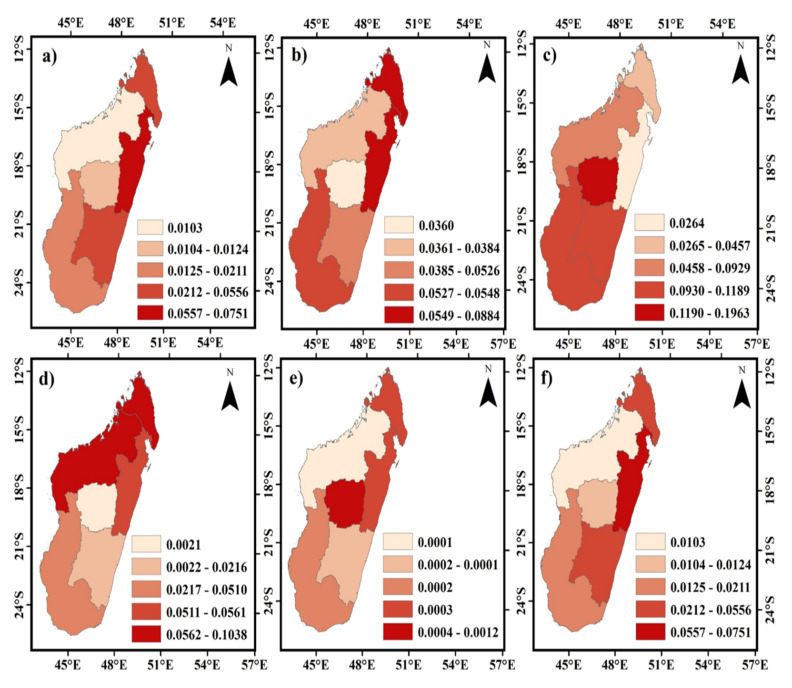
Spatial pattern of sensitivity coefficients of different land-use types of provinces in Madagascar island in 2019. Important factors were (**a**) the spatial pattern of the sensitivity coefficient of cultivated land; (**b**) the spatial pattern of the sensitivity coefficient of forestland; (**c**) the spatial pattern of the sensitivity coefficient of the savannah; (**d**) the spatial pattern of the sensitivity coefficient of wetland; (**e**) the spatial pattern of the sensitivity coefficient of built up; (**f**) the spatial pattern of the sensitivity coefficient of water.

The sensitivity coefficient of the cultivated ground area was higher in the Toamasina provinces (Figure 6a). At the same time, for forest and savannah, it was also relatively higher in Toamasina, Antsiranana, and Antananarivo provinces (Figure 6b,c). Both the wetland and water area sensitivity coefficients were much higher in the Antsiranana and Mahajanga provinces than in the other provinces (Figure 6d,f). The Antananarivo province was the most common location where built-up land sensitivity indexes were much higher than the other provinces (Figure 6e).

**Figure 7:**
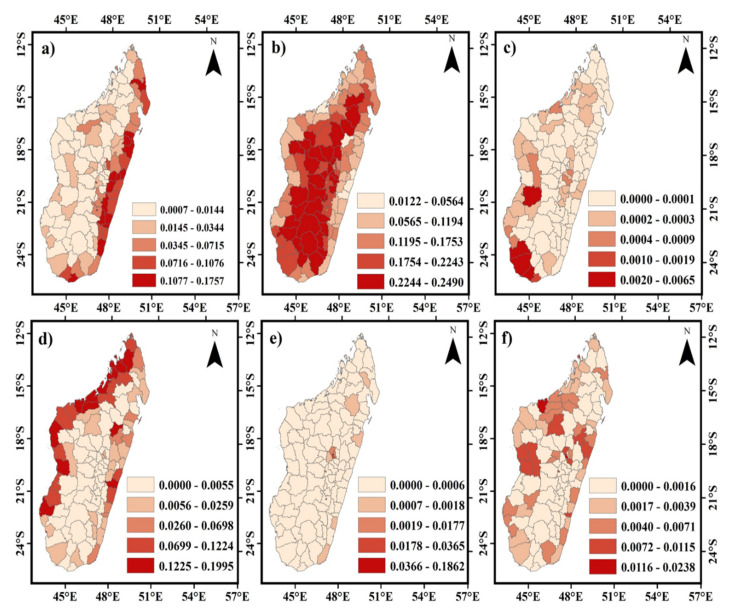
Spatial pattern of sensitivity coefficients of different land-use types of towns in Madagascar island in 2019. Important factors were (**a**) the spatial pattern of the sensitivity coefficient of cultivated land; (**b**) the spatial pattern of the sensitivity coefficient of forestland; (**c**) the spatial pattern of the sensitivity coefficient of the savannah; (**d**) the spatial pattern of the sensitivity coefficient of wetland; (**e**) the spatial pattern of the sensitivity coefficient of built up; (**f**) the spatial pattern of the sensitivity coefficient of water.

On the town scale, the sensitivity coefficient of cultivated land was higher in Sambava, Ambatondrazaka, and Tsihombe Towns (Figure 7a). In contrast, for forest and savannah, the sensitivity coefficient was relatively higher in Mandritsara, Ambalavao, Tananarivo, Mahabo, Ampanihy-Oeust, and Toliary II towns (Figure 7b,c). Both the wetland and water area sensitivity coefficients were much higher in Ambilobe, Ambanja, Nosy be hell ville, and Mitsinjo towns (Figure 7d,f). Antananarivo was the most common location with significantly higher built-up land sensitivity indexes.

## 4. Discussion

### 4.1. Influences of LULC Transformation on Ecosystem Services in Madagascar Island from 2000 to 2019

According to previous research and the current study, the most common LULC change in Madagascar is the conversion of natural vegetation to savannah. By using the estimated sizes of the seven different LULC categories and the value coefficients for ecosystem services for relevant biomes [12,55,56], it is calculated that Madagascar’s total ESV increased by 41.32 percent (US $6.99 billion) between 2000 and 2019. This positive trend was mainly caused by the growth of savannahs, which led to a rise in ecosystem services by savannahs. This increase made up for the ecosystem services reduction caused by natural ecosystems and forest loss within this time. From 2000 to 2019, farmed land, wetland, bare land, and built-up areas grew in the northern states. This growth can be linked to positive state-level changes, while growth in the southern states may be linked to more forests and wetlands. The states with a negative alter in add up to ESV declined significantly due to the loss of water bodies, principally due to a decrease in natural landscape regions in Northern Province. 

Research from other parts of the world that used an ESV assessment method similar to the present study and looked at agricultural development showed a decrease in the overall ESV. Other studies that used an ESV assessment method comparable to present research and documented agricultural expansion also revealed a decline in total ESV [57,58,59]. There could be a variety of causes for this, but one of them could be that the valuation coefficients employed were different. These studies employed different valuation coefficients than in [12], and more case studies were utilized to establish biome values [1,12,50]. The coefficient values in 2019 are ~79.88% for forests, ~95.41% for wetlands, and ~1.45% for water bodies, and more noteworthy is the valuable rise of urban ecosystem services [1]. When valuation coefficients were applied, Madagascar’s annual ESVs were much lower, and the overall ESV fell by 41.32% between 2000 and 2019. According to this, even if valuation coefficients were applied, the change path for different land cover types (excluding built-up land) was the same as in 2019. This clearly shows that expanding cultivated land reduces the value of ecosystem services, no matter what valuation coefficients are used [12].

It is essential to note the substantial ecosystem service value loss from natural ecosystems due to anthropogenic landscape substitution, even though the overall ESV grew in Madagascar island using the improved valuation coefficient during the study period [1]. In this study, conversions between natural landscapes and cultivated land from forests had a US $2.69 million net ESV loss. In this study, the forests lost US $2.69 million in net ESV due to conversions between natural landscapes and cultivated land, an increase of US $710,000 from savannahs, and a loss of US $710,000 from water bodies. The overall deterioration in the service value of Madagascar’s natural ecosystems can be mainly attributed to forest loss. It was discovered that Madagascar’s forests were the primary cause of declining ecosystem service value.

Ecosystems provide soil formation, biological control, and water, but even though they are helpful, the benefits are accompanied by declines in those ecosystem service activities. According to various studies conducted worldwide, agricultural and urban expansion negatively impact ecosystem services such as the conservation of genetic resources, erosion control, climate regulation, nutrient cycling recreation opportunities, and water regulation [60]. In Madagascar island, agricultural land use change led to more components [61]. The most important ones are waste treatment, genetic resources, food production, and habitat/refugia. They were 21.27%, 20.20%, 17.38%, and 13.80% in 2000, respectively. However, in 2019, they were 22.55%, 19.78%, 17.29%, and 13.78%, respectively. Thus, the changes that occurred during those years induced an increase in ESV value in Madagascar. While comparing the results over the year, water regulation, pollution, regulation of climate, regulation of gas, genetic resources, and leisure declined to −0.92%, −2.38%, −6.42%, −0.30%, and −1.82%, respectively. In light of Madagascar’s rapidly deteriorating climate and water management services, floods, one of the country’s most common environmental threats, has already impacted water resources, soil quality, and increased disease risk [62,63].

Despite expanding agricultural land to support the country’s rapidly growing population, food production has fallen short of projections, and food insecurity still affects more than half of the population [64,65]. In Madagascar, food insecurity may result from the country’s agricultural sector’s low production, exacerbated by several factors, including climate change, insecure land tenure, and inadequate funding [66]. Due to these factors, the sector’s labor supply has decreased, leading to a fall in agricultural productivity. Consequently, Madagascar’s vast and rising population has become dependent on imported essential goods for life [67,68]. Some of the restrictions on the agricultural sector will become even more severe as the services and natural ecosystems provided continue to deteriorate. As a result, the agricultural industry will face an increased risk of experiencing a reduction in production. The island nation of Madagascar island, which is highly susceptible to climate change effects and has a limited capacity for adaptation since its agricultural sector is utterly reliant on the natural resource base, will not be an exception [69].

In addition to the increase in Madagascar’s population, changes in land use have also been influenced by policies implemented by the government, such as those pertaining to mangroves [70]. On the other hand, policies and regulations are not always examined for compliance and fully respected throughout Africa, including Madagascar. The United Nations Framework Convention on Climate Change implemented laws all around the world on deforestation and forest degradation; however, there has been very little follow-up on these measures [71].

The sensitivity coefficient (CS) has been calculated in order to achieve an accurate and better spatial distribution pattern of different land-use types on the island. Interestingly, in our study, it ranged from 0.649 to 1.000, where forestland was the highest value, followed by wetland. Therefore, our CS is <1, which means that the ESV on the VC is inelastic [72]. Concerning the CS according to provinces, Toamasina was the highest for the cultivated ground, while Toamasina, Antsiranana, and Antananarivo provinces were the highest for forest and savannah. For wetlands and water, Antsiranana and Mahajanga were the highest among the six provinces. On the town scale, Sambava, Ambatondrazaka, and Tsihombe have had the highest CS for cultivated land. For forest and savannah, Mandritsara, Ambalavao, Antananarivo, Mahabo, Ampanihy-Ouest, and Toliary II were with the highest sensitivity coefficient. Finally, Ambilobe, Ambanja, Nosy be hell ville, and Mitsinjo were higher for wetland and water. Hence, the CS differs according to provinces and towns. Antananarivo, the capital province of the island, has the highest build-up land sensitivity indexes.

### 4.2. Policy Implications

A growing number of more severe interactions between humans and the land system are driving the compatibility of economic development with natural environment-carrying capacity. The socio-economic development was significantly out of harmony with the resource endowment and the environment’s carrying capacity due to the prolonged and rapid growth of the economy.

The government must integrate environmental protection and economic development, speed up economic restructuring, encourage industrial transformation and upgrade, and rationalize industrial function space allocation.

First, the local government must shift from prioritizing economic growth without safeguarding the natural world to balancing environmental protection with economic growth [72]. They should also recognize the importance of strengthening environmental protection to reshape the economy, shift its growth model, and pursue environmental protection development. 

Second, instead of relying solely on administrative methods to solve environmental issues, the government should employ various tools from the law, the economy, and the technology required to enforce economic and natural laws better, improve environmental protection, and create space for new development.

Third, to achieve sustainable socio-economic development with the least amount of resource consumption and environmental costs possible, the public must establish a resource-saving and environmentally friendly concept of circular economic development. They should also promote the transformation of resource utilization from a “resource–product–waste” linear mode toward a “resource–product–waste–renewable resource” circular mode. A circular economy is a more environmentally friendly kind of economic development than a traditional economy [73]. In order to make reasonable plans and actively improve organizational coordination, benefits compensation, and performance evaluation mechanisms, it is vital to consider the layout of spatial land development, spatial land carrying capacity, environmental policy, development stage, and other factors. It is also essential to design the spatial distribution of urban and agricultural space to set the flush line for lasting essential farmland security, environmental preservation, and urban advancement to investigate the mechanisms of space governance and land-use regulation [74].

An ecosystem services (ESs) evaluation helps better understand the value of natural capital. It can also serve as a reminder to decision-makers to take environmental safeguards into account while making decisions. The findings help the public realize the value that natural ecosystems bring to human beings and raise awareness of the importance of ESs. Some of the policy consequences of empirical findings may be found here. Based on statistical evidence, the urbanization level’s (UL) negative correlation with the comprehensive ecosystem services index’s (CESI) positive correlation suggests that as UL improves, (CESI) will deteriorate. There should be an increased focus on ecological, social, and economic sustainability in the future of development. At the same time, U-shaped curves between UL and CESI were also found, indicating that an increase in UL would lead to a decrease in CESI in the early stages of urbanization.

In contrast, UL would lead to an increase in CESI when the unreached a certain level [75]. A win-win outcome can be obtained when urbanization does not lead to CESI degradation. There was also a considerable impact on the dependent and independent variables in the local and outlying units. In order to protect the environment, environmental rules should not be limited to a particular entity. Variations in environmental and economic legislation caused the spillover effects. Regional collaboration and different ways of making policies are needed because regional differences must be considered when making and implementing policies in a particular area.

There is always a struggle between ideas (e.g., environmental protection, social and development, and land-use planning). Ecological implications are often overlooked in land-use planning. Multiple spatial plans in Madagascar island must be integrated to avoid undesirable effects. Agricultural, ecological, and urban designation zones and three lines (ecological, permanent basic farmland, and the urban development boundary red lines) are in place to deal with problems such as the loss of ecosystem services and the loss of farmland as construction sites grow.

### 4.3. Limitations and Potential Future Research Areas

The benefit transfer mechanism adopted in the present study has various limits. For instance, the technique assumes that the value of ecosystem services is consistent across all types of biomes/LULCs by popularizing the unit values generated out of a single place for a certain good by averaging the unit values generated in all other locations [76]. On the other hand, some services may be more useful in certain contexts than others. Once these empirical correlations between the ultimate services and ecosystem features have been established, the method is considered valid. Primary data gathering in underdeveloped nations such as Madagascar island is costly; hence, benefit transfers are frequently the only viable alternative. In [77], it is believed that future research should focus on modification of the global value coefficients to explicitly show the local ecosystem circumstances of Madagascar to maximize the use of this analysis, even though it is frequently stated that absolute precision in value coefficients is less important when measuring the direction of changes in ESV with time (as we studied). Expert surveys or statistical models of spatial and other dependencies could be used to alter these global value coefficients [78].

Future research may transfer value from a meta-analysis of analytical studies completed in African nations equivalent to Madagascar regarding their economic, social, cultural, and ecological elements to account for local income variances. This might ensure robust value based on BTM coefficients is employed in Madagascar island valuation research. Government funding promises to perform original valuation studies in Madagascar are more appropriate for obtaining location-specific ecological service values.

Measuring ecosystem services by relying on information about land use and cover and their changes in land use has proven to be a valuable tool in estimating ecosystem services and differences with land use [79]. Still, there are certain limits to land-cover datasets. Furthermore, past research has shown that the series employed as proxies for the LULC types are not necessarily ideal matches, and the land-cover databases lack precise classification [47]. For example, the datasets did not differentiate between rural and urban areas. Due to this, we computed the total built-up area ESV, which includes both urban and rural areas in Madagascar island as a proxy for urban instead of just the urban areas. This means that this land class may contain cropland and cultivated pastures in some regions, which may provide some overestimation of the ESV of this LULC type. In addition, this may show some underestimation of the ESV in other locations.

## 5. Conclusions

This study assessed the island of Madagascar’s ecosystem service values from 2000 to 2019 using ESA CCI products of land cover. The outcomes demonstrate that land cover dynamics have shifted dramatically due to rapid population growth and land urbanization. From 2000 to 2019, forest land decreased from 25.82% to 24.98%, with a difference of 0.84%. It shows a dramatic increase in urban land following the year 2000. Therefore, urban evolution is one of Madagascar’s most prominent contributing elements causing the loss of ecosystem service values. The water in the research region has shrunk significantly. Over the entire period, the estimated total ecosystem service values decreased.

Notably, the ESV lost from 2000 to 2019 was elevated to 6.99 million USD, corresponding to 41.32% due to land-use change in Madagascar island. However, the increasing economic value of farmed land can make up for this loss, making land-use changes appear economically advantageous. The decline in services such as climate regulation and water regulation, which may result in significant economic losses due to climate change and flooding, may be caused by the loss of services mostly provided by natural ecosystems over the long run. When this occurs, the gains that appear to come from increasing the amount of farmed land are also lost in a nation that is extremely vulnerable to climate change and frequently experiences natural catastrophes such as flooding. One of the most effective ways to accomplish this is through measures such as enhancing farming technology, securing land ownership to encourage farmers to grow effectively, and technological innovation.

The ESV could include in the government policy program so that the government can prioritize this matter because it is a critical study for efficiently managing our land resources. In fact, the decrease in ecosystem service values in Madagascar represents a threat that might result in an increase in the savannah and a decrease in forest and water. As an island, managing land use and land cover is an utmost task. This study recommends strategies and policy initiatives considering ecosystem-based methods to sustain equality between development steps and ecosystem health. It should be noted that ESA CCI land cover products served as the source for land cover dynamics, and it was from these datasets that our analytical results were generated. When looking at ecosystem services on a provincial scale, the results of assessments of high-resolution remotely sensed data could be very helpful. Overall, this study offers valuable information that will help in ecosystem management in Madagascar for the next decades.

## Figures and Tables

**Table 1:** The classification of Madagascar’s land cover.

Land-Cover Types	The E.S.A. C.C.I. Land Cover Maps’ Codes and Types	
Cultivated land	10	Land to grow harvested crops, non-irrigated land
	11	Herbs favorable areas
	30	Mosaic cropland (>50%)/natural vegetation (tree, shrub, herbaceous cover) (<50%)
	40	Mosaic natural vegetation (tree, shrub, herbaceous cover) (>50%)/cropland (<50%)
Forest	50	Tree cover, broadleaved, evergreen, closed to open (>15%)
	60	Tree cover, broadleaved, deciduous, closed to open (>15%)
	61	Tree cover, broadleaved, deciduous, closed (>40%)
	62	Tree cover, broadleaved, deciduous, open (15–40%)
	100	Mosaic tree and shrub (>50%)/herbaceous cover (<50%)
	110	Mosaic herbaceous cover (>50%) tree and shrub (<50%)
Shrub	120	Land covered by small bushes and trees
	122	Deciduous shrubland
Grass	130	Meadow
Wetland	170	Tree cover, flooded, saline water
	180	Shrub or herbaceous cover, flooded, fresh/saline/brackish water
Built-up area	190	Urban regions
Desert area	150	Scanty vegetation (<15%)
	200	Deserts
Water area	210	Water bodies

Important factors were adapted from the ESA CCI land cover.

**Table 2:** ESV coefficients (US$ha^−1^ 1 yr^−1^) of the corresponding biomes for Madagascar Island LULC types [1].

Service Type	Sub-Type	CuL	FoS	SaH	WeL	WaB	BiU	BaL
Provisioning	Food production	2323	200	1192	952	106	0	0
	Raw materials	219	84	54	416	0	0	0
Regulating	Gaz regulation	0	12	9	0	0	0	0
	Climate Regulation	411	2044	40	200	0	905	0
	Disturbance regulation	0	66	0	4596	0	0	0
	Water regulation	0	8	3	1789	7514	16	0
	Water supply	400	27	60	959	1808	0	0
	Waste treatment	397	120	75	111,345	918	0	0
Supporting	Control of erosion	107	337	44	3507	0	0	0
	Formation of soil	532	14	2	0	0	0	0
	Cycling of nutrient	0	3	0	577	0	0	0
	Pollination	22	30	35	0	0	0	0
	Control of the biology	33	11	31	303	0	0	0
	Habitat/refugia	0	39	1214	12,452	0	0	0
	Resources that are genetic	1042	1517	1214	243	0	0	0
Recreation	Recreation	82	867	26	2199	2166	5740	0
and culture	Cultural	0	2	167	636	0	0	0
Total Value of Ecosystem		5568	5381	4166	140,174	12,512	6661	0

Important factors “Cultivated land, forests, savannah, Wetland, water bodies, built-up land, and bare land” represent CuL, FoS, SaH, WeL, WaB, BiU, and BaL, respectively.

**Table 3:** Madagascar island LULC change (sq. ha) conversion matrix from 2000 to 2019.

10^3^	Bare Land	Built-Up	Cultivated Land	Forest	Savannah	Water Bodies	Wet Land	Total 2019	%Year 2019	Direction Change
Bare land	396.61		0.05	2.19	5.64	4.28	0.32	409.09	(0.69)	↑
Built-up	0.14	34.62	2.80	0.26	10.48	0.28	0.70	49.29	(0.08)	↑
Cultivated land	0.07	0.00	7582.83	576.83	100.78	8.56	1.80	8270.89	(13.92)	↑
Forest	0.06	0.00	165.97	14,102.27	564.44	10.61	1.39	14,844.74	(24.98)	↓
Savannah	0.63	0.00	197.86	648.18	33,890.31	7.33	3.30	34,747.61	(58.46)	↑
Water bodies	2.04		0.95	2.04	9.64	415.15	2.57	432.39	(0.73)	↓
Wetland	0.39	0.00	2.47	13.43	7.66	41.81	614.86	680.63	(1.15)	↑
total2000	399.95	34.62	7952.95	15,345.21	34,588.95	488.01	624.94	59,434.64		
% year 2000	0.67	0.06	13.38	25.82	58.20	0.82	1.05			

Important factors were the percentages of each LULC type for the corresponding years shown in the parenthesis numbers.

**Table 4:** Madagascar’s Ecosystem services value (ESV) by land-use type and changes in 2000.

The Year 2000 (10^9^ $)	Provinces	Bare Land	Built-Up	Cultivated Land	Forest	Savannah	Water Bodies	Wetland	Grand Total
1	Antananarivo	0	0.05	1.36	3.62	21.11	0.19	0.25	26.59
2	Antsiranana	0	0.03	5.73	9.97	5.76	0.31	10.61	32.42
3	Fianarantsoa	0	0.03	11.53	11.48	24.36	0.31	4.11	51.82
4	Mahajanga	0	0.04	4.63	18.62	42.95	2.49	44.62	113.35
5	Toamasina	0	0.03	13.45	18.07	5.04	0.81	9.77	47.17
6	Toliary	0	0.06	7.57	20.81	44.86	1.74	17.09	92.12

**Table 5:** Madagascar’s Ecosystem services value (ESV) by land-use type and changes in 2019.

The Year 2019 (10^9^ $)	Provinces	Bare Land	Built-Up	Cultivated Land	Forest	Savannah	Water Bodies	Wetland	Grand Total
1	Antananarivo	0	0.13	1.32	3.84	20.92	0.21	0.22	26.64
2	Antsiranana	0	0.00	5.94	9.50	5.97	0.27	10.97	32.65
3	Fianarantsoa	0	0.00	11.58	10.97	24.70	0.31	4.51	52.08
4	Mahajanga	0	0.00	4.82	17.93	43.35	2.13	48.43	116.65
5	Toamasina	0	0.00	14.45	17.02	5.09	0.73	10.80	48.10
6	Toliary	0	0.00	7.92	20.61	44.72	1.53	19.18	93.97

**Table 6:** Madagascar’s Ecosystem services value (ESV) by land-use type and changes from 2000 to 2019.

ESV	Cultivated Land	Forest	Savannah	Wetland	Built-Up	Bare Land	Water Bodies	Total
2000($) ×10^9^	44.28	82.57	144.09	87.60	0.23	0.00	6.11	364.88
2000 (%)	12.14	22.63	39.49	24.01	0.06	0.00	1.67	100.00
2019($) ×10^9^	46.05	79.88	144.80	95.41	0.33	0.00	5.40	371.87
2019 (%)	12.38	21.48	38.94	25.66	0.09	0.00	1.45	100.00
Change 2000–2019 ($)	1.77	−2.69	0.71	7.81	0.10	0.00	−0.71	6.99
Change 2000–2019 (%)	4.00	−3.26	0.49	8.92	42.74	0.00	−11.56	41.32

**Table 7:** Estimated values for diverse ecosystem functions ( $ESV_{f}$) in Madagascar island from 2000 to 2019.

		2000		2019		2000–2019	
		(10^9^ USD)	%	(10^9^ USD)	%	(10^9^ USD)	%
Provisioning	PRODUCT	63.42	17.38	64.30	17.29	0.87	1.38
	Raw materials	5.16	1.41	5.22	1.40	0.06	1.15
Regulating	Regulation of gas	0.50	0.14	0.49	0.13	0.00	−0.92
	Regulation of climate	36.17	9.91	35.31	9.50	−0.86	−2.38
	Regulation of disturbance	3.89	1.06	4.11	1.10	0.22	5.74
	Regulation of water	5.01	1.37	4.69	1.26	−0.32	−6.42
	Supply of water	7.15	1.96	7.23	1.94	0.08	1.06
	Treatment of waste	77.63	21.27	83.85	22.55	6.23	8.02
Supporting	Control of erosion	9.74	2.67	9.80	2.64	0.07	0.69
	Formation of soil	4.51	1.24	4.68	1.26	0.16	3.60
	Cycling Nutrient	0.41	0.11	0.44	0.12	0.03	7.53
	Pollination	1.85	0.51	1.84	0.50	0.00	−0.13
	Biological control	1.69	0.46	1.72	0.46	0.03	1.58
	Habitat/refugia	50.37	13.80	51.24	13.78	0.87	1.72
	Genetic resources	73.71	20.20	73.49	19.76	−0.22	−0.30
Recreation and Culture	Recreation	17.49	4.79	17.17	4.62	−0.32	−1.82
	Cultural	6.20	1.70	6.27	1.68	0.06	0.98
	TOTAL	364.89	100	371.83	100		

**Table 8:** Sensitivity coefficient resulting from an adjustment of the equivalent value coefficient.

Land-Use Types	2000 CS	2005 CS	2010 CS	2015 CS	2019 CS
Cultivated land VC ± 50%	0.109	0.107	0.108	0.109	0.112
Forestland VC ± 50%	0.649	0.635	0.634	0.631	0.643
Savannahs VC ± 50%	0.010	0.004	0.001	0.001	0.002
Wetland VC ± 50%	0.216	0.240	0.244	0.245	0.230
Built up VC ± 50%	0.001	0.001	0.001	0.001	0.001
Bare land VC ± 50%	0.000	0.000	0.000	0.000	0.000
Water bodies VC ± 50%	0.015	0.014	0.013	0.013	0.013

Important factors were 0.00, demonstrating that the esteem is very little and exceptionally near zero. CS: coefficient of sensitivity.

## Data Availability

The link for the data is provided within the paper and references.
